# Slowly Progressive Pulmonary Metastasis From Melanoma Mimicking Adenocarcinoma In Situ

**DOI:** 10.1016/j.atssr.2025.07.023

**Published:** 2025-08-26

**Authors:** Kaito Yano, Mikito Suzuki, Tomohiro Imoto, Reiko Shimizu, Kazuo Nakagawa

**Affiliations:** 1 Department of Thoracic Surgery, Tokyo Metropolitan Cancer and Infectious Diseases Center Komagome Hospital, Tokyo, Japan

A 67-year-old man underwent wide local excision and prophylactic lymphadenectomy for a right thigh malignant melanoma (stage IIB). An annual computed tomography scan 10 years after the surgery revealed a 10-mm ground-glass nodule (GGN) in the right upper lobe (Figure 1A, yellow arrow). An approximately 7-mm GGN was identified 24 months prior, although high-resolution computed tomography was not available (Figure 1B, yellow arrow). Wedge resection was performed on the basis of the presumptive diagnosis of adenocarcinoma in situ (AIS). The resected lesions exhibited black pigmentation (Supplemental Figure). On histologic evaluation, atypical pigmented cells were spread along the alveolar septa and were accompanied by stromal thickening (Figure 2). Immunohistochemistry revealed diffuse Melan-A expression, confirming the diagnosis of metastatic melanoma.

**Figure 1:**
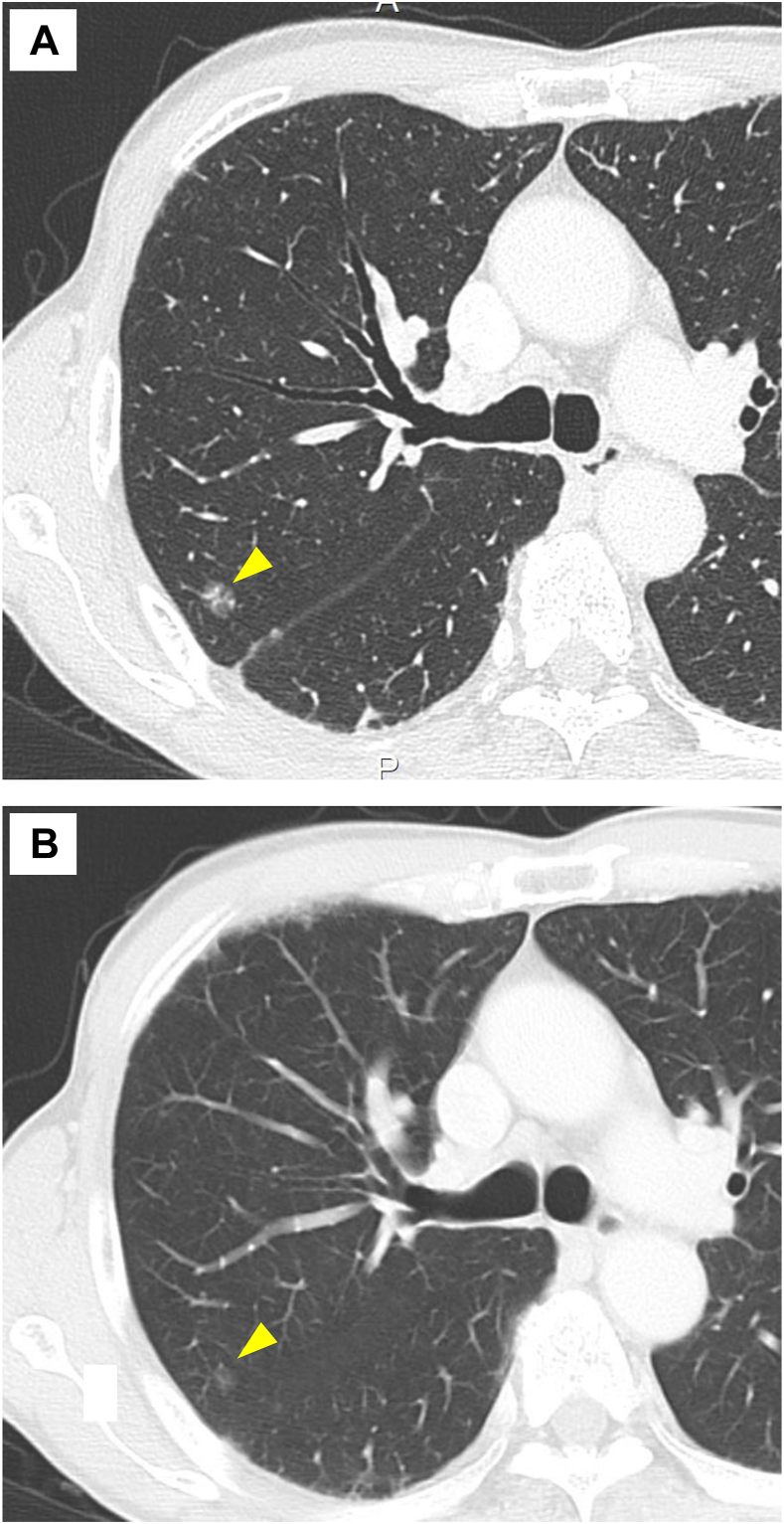

**Figure 2:**
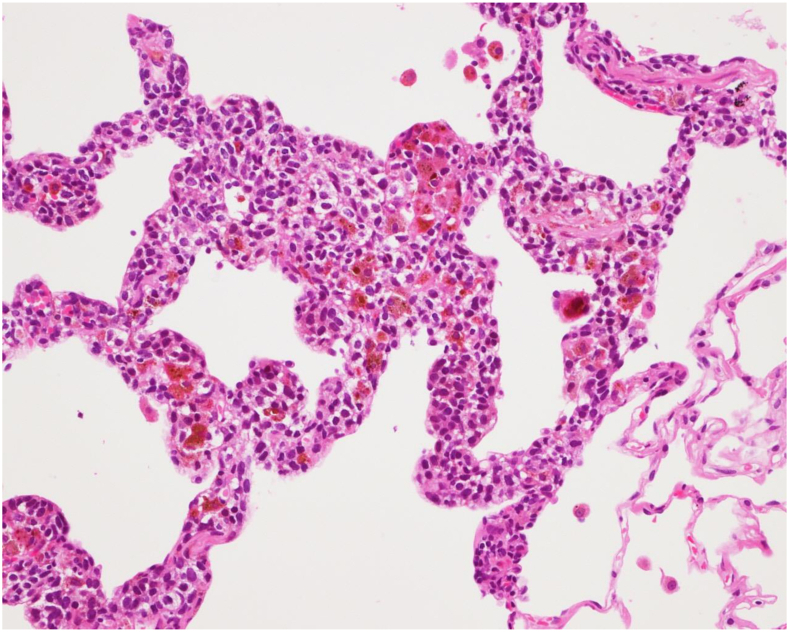

Pulmonary metastases (PMs) presenting with GGN are rare; however, 4.6% of PMs from melanoma can be manifested as GGNs, although solitary cases are limited.^1^ The lepidic growth of the tumor cells led to this radiographic appearance. The reported mean tumor doubling time for PMs from melanoma with GGN appearance is 52 days, which is useful for differentiating them from AIS of 813 days.^1^ In this case, the GGN displayed extremely slow growth, making differentiation from AIS challenging. In patients with history of melanoma, slowly progressive small GGNs with controversial surgical indications should be considered for resection considering the possibility of PMs.

## Figures and Tables

**Supplementary Figure 1:**
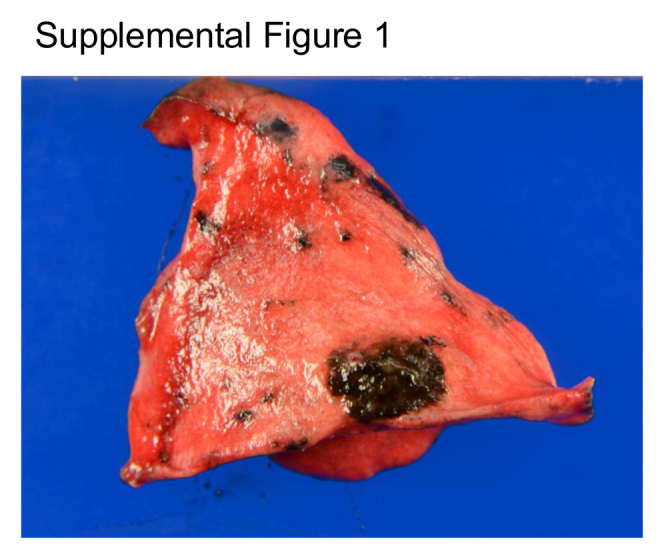


## References

[1] Todoroki K., Kawakami S., Kiniwa Y. (2025). Pulmonary metastases from malignant melanoma showing ground-glass opacity nodules. Jpn J Radiol.

